# Neuronal–Glial Interaction in a Triple-Transgenic Mouse Model of Alzheimer’s Disease: Gene Ontology and Lithium Pathways

**DOI:** 10.3389/fnins.2020.579984

**Published:** 2020-12-01

**Authors:** Nicole Kemberly R. Rocha, Rafael Themoteo, Helena Brentani, Orestes V. Forlenza, Vanessa De Jesus Rodrigues De Paula

**Affiliations:** ^1^Laboratório de Psicobiologia (LIM23), Departamento e Instituto de Psiquiatria, Hospital das Clinicas HCFMUSP, Faculdade de Medicina, Universidade de São Paulo, São Paulo, Brazil; ^2^Laboratorio de Neurociencias (LIM27), Departamento e Instituto de Psiquiatria, Hospital das Clinicas HCFMUSP, Faculdade de Medicina, Universidade de São Paulo, São Paulo, Brazil

**Keywords:** lithium, gene ontology, triple-transgenic model of Alzheimer’s disease, hippocampus, neuronal-glial interaction

## Abstract

Neuronal-glial interactions are critical for brain homeostasis, and disruption of this process may lead to excessive glial activation and inadequate pro-inflammatory responses. Abnormalities in neuronal-glial interactions have been reported in the pathophysiology of Alzheimer’s disease (AD), where lithium has been shown to exert neuroprotective effects, including the up-regulation of cytoprotective proteins. In the present study, we characterize by Gene Ontology (GO) the signaling pathways related to neuronal-glial interactions in response to lithium in a triple-transgenic mouse model of AD (3×-TgAD). Mice were treated for 8 months with lithium carbonate (Li) supplemented to chow, using two dose ranges to yield subtherapeutic working concentrations (Li1, 1.0 g/kg; and Li2, 2.0 g/kg of chow), or with standard chow (Li0). The hippocampi were removed and analyzed by proteomics. A neuronal-glial interaction network was created by a systematic literature search, and the selected genes were submitted to STRING, a functional network to analyze protein interactions. Proteomics data and neuronal-glial interactomes were compared by GO using ClueGo (Cytoscape plugin) with *p* ≤ 0.05. The proportional effects of neuron-glia interactions were determined on three GO domains: (i) *biological process*; (ii) *cellular component*; and (iii) *molecular function*. The gene ontology of this enriched network of genes was further stratified according to lithium treatments, with statistically significant effects observed in the Li2 group (as compared to controls) for the GO domains *biological process* and *cellular component*. In the former, there was an even distribution of the interactions occurring at the following functions: “positive regulation of protein localization to membrane,” “regulation of protein localization to cell periphery,” “oligodendrocyte differentiation,” and “regulation of protein localization to plasma membrane.” In *cellular component*, interactions were also balanced for “myelin sheath” and “rough endoplasmic reticulum.” We conclude that neuronal-glial interactions are implicated in the neuroprotective response mediated by lithium in the hippocampus of AD-transgenic mice. The effect of lithium on homeostatic pathways mediated by the interaction between neurons and glial cells are implicated in membrane permeability, protein synthesis and DNA repair, which may be relevant for the survival of nerve cells amidst AD pathology.

## Introduction

Alzheimer’s disease (AD) is the most prevalent age-related neurodegenerative disease, affecting more than 30 million individuals worldwide. The disease causes progressive cognitive decline and neuropsychiatric symptoms that ultimately lead to functional disability and dementia (Qiu et al., 2009). Functional and structural changes to the hippocampus occur since early stages of AD pathogenesis, and this process leads to progressive deficits in memory and learning (Mak et al., 2017; Weiner et al., 2017). According to the amyloid hypothesis of AD (Selkoe and Hardy, 2016), the overproduction of insoluble forms of the amyloid-beta peptide (Aβ) leads to its extracellular accumulation in the form of senile/neuritic plaques, with subsequent noxious effects on neuronal homeostasis. In particular, damage to the cytoskeleton, ultimately leading to the formation of neurofibrillary tangles (NFT), are largely dependent on the hyperphosphorylation of tau protein, causing dystrophy of neurites and synaptic damage (Lovestone and Reynolds, 1997). This process is largely accompanied by inflammatory changes, including activated microglia and reactive astrocytes (Farfara et al., 2008; Bedner et al., 2015; Hemonnot et al., 2019; Sharoar et al., 2019).

Reactive astrocytes are known to upregulate Aβ production by increasing the expression of β- and γ-secretases (Frost and Li, 2017). Activated microglia stimulate phagocytic activity, release chemokines and neurotrophic factors, and present T-cell antigens (Hanisch and Kettenmann, 2007). The systemic immune response may indirectly affect certain neural processes—some of which are disrupted by AD pathogenesis—by the recruitment of monocytes and subsequent modifications in tissue-resident microglia in the central nervous system (Lai and McLaurin, 2012; Guillot-Sestier et al., 2015; Jay et al., 2015; Koronyo et al., 2015; Matcovitch-Natan et al., 2016; Deardorff and Grossberg, 2017). Studies have shown that, under specific conditions, microglial cells express pro-inflammatory activity, which may hasten the progression of the ongoing disease (Heppner et al., 2015; Wang et al., 2015). The systemic immune response may indirectly affect certain neural processes—some of which are disrupted by AD pathogenesis—by the recruitment of monocytes and subsequent modifications in tissue-resident microglia in the central nervous system (Lai and McLaurin, 2012; Guillot-Sestier et al., 2015; Jay et al., 2015; Koronyo et al., 2015; Matcovitch-Natan et al., 2016; Deardorff and Grossberg, 2017). Control mechanisms, while essential for neuronal-glial function to ensure their immune activation, can become noxious in situations where strong phagocytic activity is needed, as observed in the aging brain and in neurodegenerative conditions. Lithium salts have long been used for the treatment of major psychiatric disorders, although its exact mechanisms of action remain unclear.

Lithium modifies multiple signaling pathways, most importantly those related to the expression (Mendes et al., 2009) and activity (Forlenza et al., 2012) of glycogen synthase kinase-3β (GSK3β), a highly expressed kinase in neurons and glial cells. GSK3β modulates key homeostatic processes, including pro-survival responses, apoptosis, and neurodevelopmental and differentiation pathways (Hampel et al., 2019). GSK3β has been centrally implicated in AD pathogenesis (Forlenza et al., 2014; Kerr et al., 2018; Hampel et al., 2019), affecting both the overproduction of the amyloid-beta peptide (Aβ) and hyperphosphorylation of microtubule-associated protein Tau, mechanisms that respectively lead to the formation of senile plaques and neurofibrillary tangles—pathological hallmarks of AD (Tan et al., 2010; Forlenza et al., 2014; Kerr et al., 2018; Hampel et al., 2019; Li et al., 2020). Lithium is a potent inhibitor of GSK3β activity (Engel et al., 2006; Tan et al., 2010; Forlenza et al., 2014; Kerr et al., 2018; Hampel et al., 2019; Li et al., 2020) and, in animal models of AD, chronic lithium treatment was associated with reduced hyperphosphorylation of Tau and Aβ accumulation (Su et al., 2004; Noble et al., 2005; Engel et al., 2006; Forlenza et al., 2014). Clinical extrapolations of these effects are scarce, but the available evidence from a small number of clinical trials seem to be in line with experimental findings (Forlenza et al., 2011; Ladeira et al., 2013). There is also evidence of the neuroprotective effects of lithium, including increased neuronal survival by overexpression of p53 and Bcl-2 genes, stimulation of trophic responses via up-regulation of brain-derived neurotrophic factor (BDNF) (de Sousa et al., 2011; De-Paula et al., 2016a) and vascular endothelial growth factor (VEGF) (Guo et al., 2009), modulation of membrane homeostasis (De-Paula et al., 2015), reduction of pro-inflammatory cytokines IL-1β and TNF-α (De-Paula et al., 2016b), and prevention of oxidative cellular damage by activation of nuclear factor erythroid 2-related factor 2 (Nrf2) (Alural et al., 2015).

Neuron-glia interactions may also be important to the regulation of anti-inflammatory and antioxidant responses in the neural milieu, as well as to the actual survival of both cell types. Experimental models have also shown that overactive GSK3β is also associated with reactive astrocytosis and microgliosis, an effect that may also be attenuated by lithium (Tang et al., 2016). In a recent study conducted by our group, we showed that that chronic treatment with lithium chloride at subtherapeutic concentrations was able to modify the secretion of pro- and anti-inflammatory interleukins in co-cultures of cortical and hippocampal neurons with glial cells, and this effect was associated with the preservation of culture integrity (De-Paula et al., 2016b; Goshi et al., 2020). Gene Ontology (GO) is a bioinformatics approach to ascertain and to unify the representations of gene- and gene product attributes across species, focusing on the related functions of these interactions. In brief, GO summarizes our current knowledge on biological domains regarding three main aspects: (i) *biological process*, (ii) *molecular function*, and (iii) *cellular component*—all attributes of genes, gene products or gene-product groups. In this context, to explore the molecular aspects underlying the complex changes observed in AD, a number of genome-wide expression profiling experiments were performed in hippocampal tissues from AD patients (Elsheikh et al., 2019; Andrews et al., 2020), including functional pathway enrichment analyses with gene ontology terms of expression profiling. The most relevant GO categories in *molecular function* are those primarily involved in synaptic function, notably “synaptic signaling, transmission and processing,” and “synaptic vesicle metabolism” (Wu et al., 2019). As for *cellular component*, relevant GO categories were those related to “synapse part,” “neuron part,” “synapse,” “pre-synapse,” “exocytic vesicle,” “transport vesicle,” “cytoplasmic, membrane-bounded vesicle,” “synaptic vesicle,” “secretory vesicle,” “neuron projection,” “exocytic vesicle membrane,” “synaptic vesicle membrane,” “post-synapse,” “transport vesicle membrane,” “cell junction,” “cytoplasmic vesicle part,” and “excitatory synapse.” For *biological process*, relevant GO categories were implicated in “synaptic signaling,” “anterograde trans-synaptic signaling,” “trans-synaptic signaling,” “chemical synaptic transmission,” and “cell–cell signaling” (Wu et al., 2019).

We hypothesize that the study of ontological pathways can help elucidate the importance of neuron-glia interactions in processes related to the maintenance of neural homeostasis in the hippocampus, particularly in the presence of AD pathology. The aim of the present study is to determine the profile of neuronal-glial interactions in the biological pathways of proteins in the hippocampal tissue from AD-transgenic mice, and to further investigate the effects of lithium on these interactions. To pursue these goals, we designed a combined *in silico*/*in vivo* approach based on the following steps: (i) literature search; (ii) STRING analysis (characterization of network interactions); (iii) *in vivo* experimentation (lithium intervention in transgenic and wild-type mice); (iv) proteomics; (v) integrated analysis and gene ontology.

## Materials and Methods

### Literature Search

A systematic literature search was conducted in PubMed database using the mesh terms “neuron,” “glia,” and “interactions,” “cell culture,” “gene interactions,” “lithium.” We addressed scientific articles published in English between 2013 and April 11, 2019. Eligible papers were included if based on *in vitro* (tissue culture) or *in vivo* (animal model) studies evaluating neuron-glia gene interactions through analysis of DNA or RNA, and further addressing the influence of lithium on these interactions. Papers would be excluded if based on studies conducted in human samples, or if reported data from protein analysis only. The literature search procedures were: descriptor search; evaluation of article titles; abstract reading; detailed assessment of “Materials and Methods” section; article selection according to inclusion/exclusion criteria; and secondary search through analysis of reference lists in selected studies. Eighty potentially relevant articles were retrieved from literature search, with focus on the following topics: neuron-glia interactions in cell cultures (50 articles); neuron-glia gene interactions (25 articles); neuron-glia interactions plus lithium (4 articles); lithium influence on neuronal glia (1 article); and lithium influence on neuron-glia interactions (0 articles in).

### Characterization of Network Interactions (STRING)

Selected genes from literature search were submitted to STRING, a web-based tool dedicated to the analysis of functional protein association networks (available at https://string-db.org/). An “enriched network” was then obtained within this database of documented and predicted PPIs, including computational prediction of direct (physical) and indirect (functional) associations by aggregating interactions from other (primary) databases. Three interactome databases were consulted to build PPI networks: HPRD (Keshava Prasad et al., 2009), MINT (Licata et al., 2012), and Intact (Kerrien et al., 2012). A cut-out of this network was done comprising up to the second neighbors from the genes selected from the literature.

### *In vivo* Experiments

#### Animal Model

All animal experiments were approved by the local ethics committee (CAPPesq n°1293/09) in accordance with Directive 2010/63/EU. Fifty-three young-adult (3-month old) male mice were purchased from Jackson Laboratory: of these, 27 were triple-transgenic AD (3×-TgAD), namely B6; 129-Psen1tm1MpmTg (APPSwe, tauP301L) 1Lfa/Mmjax, and 26 were wild-type (Wt) mice (B6129SF2/J). Mice were kept under controlled environment at the Central Animal Facility at the Faculty of Medicine, University of São Paulo (FMUSP), housed in standard cages (four or five animals per cage) sized 41 × 34 × 16cm (length, width, height) and kept at controlled temperature (22 ± 1°C), relative humidity (50–60%) and 12-h light/dark cycle.

#### Treatment Groups

Lithium-supplemented chow was weekly prepared by Nutri Experimental^®^ (Brazil), by mixing lithium carbonate (Li_2_CO_3_; Merck) to regular chow. 3×-TgAD and Wt mice were evenly distributed into six subgroups: (1) Li0, 3×-TgAD (*n* = 9) or Wt (*n* = 9) mice fed with regular chow (treatment control groups); (2) Li1, 3×-TgAD (*n* = 9) or Wt (*n* = 9) mice fed with 1.0 g of Li_2_CO_3_/kg of chow; (3) Li2, 3×-TgAD (*n* = 9) or Wt (*n* = 8) mice fed with 2.0 g Li_2_CO_3_/kg of chow. All animal had access to food and water *ad libitum*. Lithium-treated mice also received a bottle with 0.9% NaCl for *ad libitum* intake, according to previous experience in our lab (Cardillo et al., 2018). Treatments lasted for 34 weeks, until animals reached 11 months of age, when it is accepted that mice become middle aged. Animal weight was measured monthly throughout the experiment. Forty animals reached the endpoint, being 18 3×-TgAD and 22 Wt. Thirteen mice (24.5% of the initial sample) died during the intervention phase; of these, nine were transgenic and four Wt mice (30 and 15% of each initial group respectively), with an even distribution according to intervention groups (Li0, 4; Li, 5; Li2, 4). Animals were euthanized by decapitation, after which the hippocampal tissues were dissected and immediately frozen at −80°C until experimentation.

#### Measurement of Serum Lithium Concentration

Serum lithium concentrations (mM) were determined 2 days before the intervention endpoint. Blood samples were collected into heparin-coated tubes from tail veins, and the plasma was separated by high centrifugation. Lithium concentration was determined by an ion analyzer (Electrolyte Analyzer 9180, Roche). Mean serum concentrations of lithium in each group are shown in Table 1. Both lithium diets yielded mean concentrations within subtherapeutic limits, ranging from 0.11 mM (minimum value in Li1) to 0.57 mM (maximum value in Li2), whereas lithium levels were undetectable in mice fed with standard chow. Although serum lithium concentrations tended to be higher among Li2 as compared to Li1 mice, statistically significant differences were only observed when compared to Wt animals.

**TABLE 1:** Characterization of study groups according to genetic characteristics of mice and intervention.

Group	N (T0/T1)	Serum lithium (mmol/L)	T0 weight (g)	Tl weight (g)	Weight change (g)
WtLi0	9/8	–	27.30 ± 2.54	42.18 ± 5.67	14.88 ± 4.43
WtLil	9/8	0.16 ± 0.04	24.33 ± 1.66	37.79 ± 3.71	13.46 ± 4.39
WtLi2	8/6	0.26 ± 0.07	27.57 ± 2.70	37.07 ± 4.73	9.50 ± 6.74
TgLi0	9/6	–	26.97 ± 3.52	38.87 ± 5.13	11.90 ± 5.03
TgLil	9/5	0.21 ± 0.07	23.68 ± 2.47	33.36 ± 3.78	9.68 ± 3.45
TgLi2	9/7	0.35 ± 0.20	22.94 ± 3.42	34.71 ± 4.27	11.77 ± 4.55

**Wild type (Wt) and* 3×*Tg-AD (Tg) mice were allocated into experimental (lithium intervention) and comparison groups. i.e., 1 g of Li_2_CO_3_/Kg of chow (Lil); 2 g of Li_2_CO_3_/Kg of chow (Li2); or standard chow (untreated controls; Li0). N, number of mice in each group; g, grams. Animal mortality (second column) can be depicted by subtracting the number of experimental animals at endpoint (Tl) from the original number at baseline (TO). All values are presented as mean ± standard deviations (SD). Statistical analysis of the number of mice (baseline endpoint) and weight change per group (Wt vs. Tg): WtLiO/Lil/Li2 vs. TgLiO/Lil Li2. N.S.; or exposure to lithium treatment (lithium vs. non-lithium): WtLiO vs. WtLil/Li2, N.S.; TgLi0 vs. TgLil/Li2. N.S.**

A higher mortality was observed among 3×-TgAD mice, but this effect was apparently unrelated to lithium treatment. Lithium levels at the endpoint of the study were far below toxic levels (i.e., at subtherapeutic range), both in 3×-TgAD and WT mice. In addition, mortality was similar in both intervention groups (lithium vs. non-lithium), although higher among transgenic mice, presumably influenced by the genetic status rather that the exposure to lithium. Compared to those receiving standard chow, lithium-treated 3×-TgAD mice displayed higher (statistically significant) urinary volumes, which was associated with increased saline solution intake but not with weight gain (data not shown).

#### Protein Precipitation and Digestion

The protein fraction (100 μg) from hippocampal tissues was precipitated with methanol/chloroform, according to Wessel and Flügge (Wessel and Flügge, 1984). Protein pellets were resuspended in 100 mM Tris buffer, pH 8.5, containing 8 M urea, and digested with a protocol adapted from Klammer and MacCoss (Klammer and MacCoss, 2006). Briefly, disulfide bonds were reduced in 5 mM tris-(2-carboxyethyl)-phosphine-hydrochloride (TCEP) for 20 min at 37°C and then cysteines were alkylated in 25 mM iodoacetamide (IAM) for 20 min. at room temperature in the dark. Urea was diluted to 2 M with 100 mM Tris pH 8.5, and proteins were digested with trypsin in a ratio of 1:100 enzyme/protein with 1 mM CaCl2 by overnight incubation at 37°C. After overnight trypsin digestion, reaction was quenched in 2% formic acid and samples were stored at −20°C until use.

### Proteomics

#### Liquid Chromatography/Mass Spectrometry (LC-MS)

LC-MS/MS experiments were performed in a quaternary HP 1100 series HPLC pump (Agilent technology) coupled to an LTQ-Velos Orbitrap mass spectrometer (Thermo Fisher Scientific). Electrospray was performed directly from the tip of the analytical column with Solution A (5% acetonitrile and 0.1% formic acid), solution B 980% acetonitrile and 0.1% formic acid) and solution C (500 mM ammonium acetate, 5% acetonitrile and 0.1% formic acid). Flow rate was almost 300 nL/min. Biphasic MudPIT columns (150μm ID/360μm OD fused-silica capillary) were prepared in house by slurry packing and 2.5 cm of SCX resin (5μm Partisphere, Whatman) followed by 2.5 cm of C18 reversed phase resin (5μm ODS-AQ C18 Yamamura Chemical Lab). Capillary analytical columns (100 μm ID/360 μm OD capillary) were slurry packed with 20 cm of C18 reversed phase packing material behind a 5 μm ID tip.

MudPIT columns were loaded with 20μg of peptide mixture and a 10-h MudPIT separation method was used, consisting of a transfer step followed by seven separation steps (five 60-min steps, one 120-min step, and one 180-min step). The transfer step was a gradient up to 50% B over 20 min., followed by an increase to 100% B in 4 min., 100% B for 2 min. and re-equilibration with 100% A for 4 min. The five 60-min. gradients consisted of 2 min. of salt injection (10, 20, 30, 50, and 70% C) followed by 2 min. of 100% A, a linear gradient from 0 to 40% B over 45 min and re-equilibration in 100% A for 10 min. The 120-min gradient was 2 min of 100% C followed by 2 min of 100% A, a linear gradient from 0 to 50% B over 85 min, an increase to 100% B over 15 min, 100% B for 3 min and re-equilibration in 100% A for 12 min. The final 180-min gradient consisted of 5 min of 90% C/10% B followed by 4 min of 100% A, 0 to 10% B in 15 min, 10 to 20% B in 60 min, 20 to 50% B in 60 min, 15 min up to 100% B, 100% B for 10 min and re-equilibration in 100% A for 10 min. LTQ-Velos Orbitrap was operated in a dataset dependent mode. Full MS1 scans were collected in the Orbitrap (300–1,200 m/z range, 60 K resolution and AGC target of 5 × 105) and the 20 present in large quantities ions per scan were selected for CID MS2 in the ion trap (intensity of 500 and AGC target of 1 × 104). Maximum fill times were 250 and 100ms for MS1 and MS2 scans, respectively. Exclusion was enabled with repeat count of 1 with duration of 150s, exclusion sample size of 500 and exclusion duration of 120 s.

#### Characterization of Proteins

Peptide and protein identification were carried out in Integrated Proteomics Pipeline—IP2 (Integrated Proteomics Applications, Inc.)^1^. Results from Tandem mass spectra were extracted from RAW files using RawXxtract 1.9.9.2 (Gene replacement and quantitative mass spectrometry approaches validate guanosine monophosphate synthetase as essential for Mycobacterium tuberculosis growth—ScienceDirect) and searched with ProLuCID v.1.3.1 (Wessel and Flügge, 1984) against a *Mus musculus* reference proteome database from UniProtKB and reverse sequences. Search dataset included fully and half-tryptic peptide candidates, using carbamidomethylating of cysteine as static modification. Dataset was searched with 50 ppm ion tolerance and 600 ppm fragment precursor ion tolerance. Peptide candidates were filtered using DTASelect 2.0 (Klammer and MacCoss, 2006; Villela et al., 2015), with a peptide-spectra match mass variation <10 ppm, a minimum of two peptides per protein and a false discovery rate of 1% related on the number of decoys.

#### Protein-Protein Interactions (PPI)

To assess the difference in co-expression of the differentially expressed in the proteomics, we performed an analysis of gene co-expression networks (Silva et al., 2012). The correlation of each gene and its partners was calculated by Pearson’s Coefficient of Correlation (PCC). Differences in PCC between groups were compared to 1,000 lists of co-expression differences mixing cases and controls, that is, 1,000 random sets (*p* ≤ 0.05). We also used the WGCNA (Weighted Correlation Network Analysis) tool (Langfelder and Horvath, 2008) to search for co-expression module. The network was viewed using Cytoscape (Cline et al., 2007), and we analyzed the differences topological properties of gene networks seeking to prioritize AD-related partners and to compare findings in the hippocampus. For identification of broker and bridge genes (Son et al., 2003; Aluise et al., 2010), we used the Interactome Graph website^2^. The data was analyzed in software R and Cytoscape (v 3.2), comparing lithium-treated groups (Li1, Li2) and controls (Li0) with gene ontology.

### Gene Ontology

Proteomics and neuron-glia interaction were compared by Gene Ontology pathways using ClueGO, a Cytoscape plug-in that visualizes non-duplicated biological terms for major clusters of genes in a functionally network. We used ClueGO to perform single cluster analyses and comparisons of numerous clusters (lists of genes) using the following basic parameters for *Mus musculus* (definition of organism): we used “hyper-geometric test” for statistical testing, and “Benjamini and Hochberg False Discovery Rate” (setting *p* ≤ 0.05) for test correction; and “biological process,” “cellular component,” and “molecular function” were used for ontology files.

## Results

### Characterization of a Hypothetical Neuron-Glia Interaction Network Based on Literature Search and STRING

Based on the review of 80 potentially relevant articles, 56 were regarded unrelated to the objectives of the present study, and therefore excluded from the analysis. Another 20 articles were excluded due to methodological incompatibilities, so that the final compilation comprised only four eligible studies. The main characteristics of these studies and the respective genes that were identified are listed in Table 2.

**TABLE 2:** Characteristics of the eligible studies selected from literature search.

References	Specie/Model	Biological matrix	Drug treatment	Experiment	Selected genes
Kangas et al. (2016)	13.5-day-old mouse embryos mice (C57BL/6J01aHsd, Harlan Holland)	Dorsal root ganglions	–	Hybridization Kit (Agilent) onto SurePrint G3 Mouse GE 8 × 60K arrays (Agilent. Design ID 028005),	*Ngfr, Pon3fl Nefm Mag, Mpz, Mbp*
Emamghoreishi et al. (2015)	18-day embryos of Sprague-Dawley rats	Embryonic cortices	1 mM lithium	PCR	S1OOβ and GAPDH
Chen et al. (2015)	Timed-pregnant Fisher 344 rats al day 14 of gestation	Cortices and midbrain	–	PCR	GDNF F2, BDNF F1, GAPDH F2, TNFalfa F4, Inos F1, INOSR1, GDNF R2
Yang et al. (2014)	PO or Pl adult male Sprague-Dawley rats	Hippocampi	–	PCR	GAPDH, ephrin A1, ephrin A2, ephrin A3, ephrin A4, ephrin A5

**FIGURE 1:**
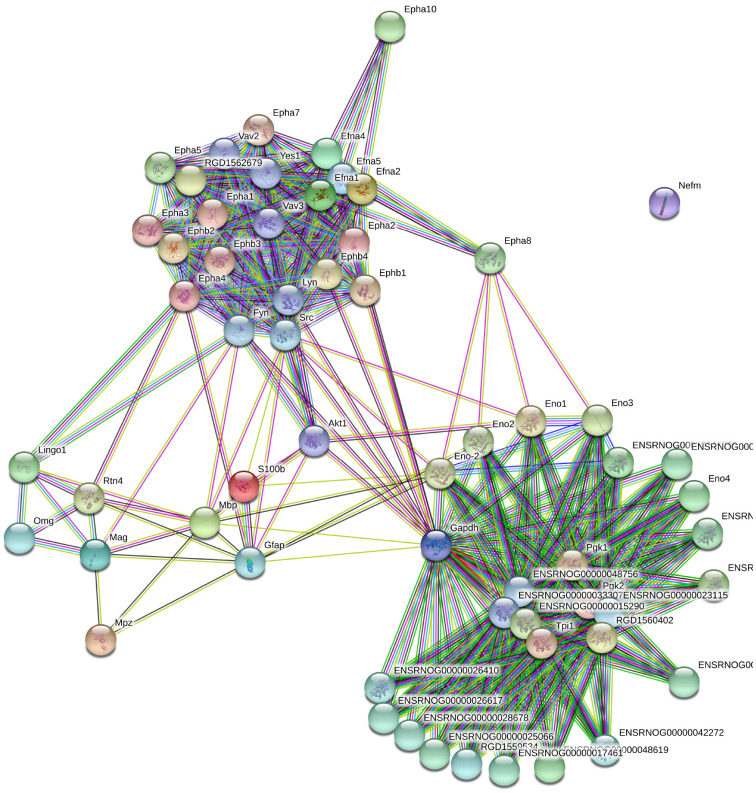
Grown network with no more than 50 interactions (https://string-db.org/).

Selected genes and respective IDs for *Mus musculus* are displayed in Supplementary Table 1. These genes were submitted to STRING and enriched with 50 interactions. The disconnected genes were excluded from the analysis. The total number of genes after de enrichment analysis was 42 (Supplementary Table 2). Figure 1 displays the interactome between the 42 final genes obtained by enrichment analysis.

### Proteomics and Neuron-Glia Interactions

Differentially expressed proteins in hippocampal proteomes from Wt and 3×-TgAD mice were analyzed in ClueGo, according to the parameters described in the “Materials and Methods” Section. Table 3 summarizes neuron-glia interactions according to the GO domains *biological process*, *cellular component*, and *molecular function*, indicating the related functions that reached statistical significance (*p* < 0.05) in distinct experimental groups (Table 3B–G). Results indicate statistically significant functional difference between untreated 3×-TgAD and Wt mice (Li0), as well as when these animals were treated with different concentrations of lithium (Li1 and Li2). Neuron-glia interactions related to GO domain *biological process* indicated the participation of genes related to transmembrane-ephrin receptor activity, glycolytic process and ephrin receptor signaling pathway. As for *cellular component*, relevant genes were associated with the “postsynaptic specialization,” “intracellular component,” and “phospho-pyruvate hydratase complex.” The *molecular function* domain was predominantly associated with “transmembrane-ephrin receptor activity,” “ephrin receptor binding,” and “neurotrophin receptor binding.” In untreated (Li0) Wt mice, we found statistically significant associations for “membrane transport” (*biological process*); “Golgi complex” (cellular component); and “catalytic activity” (molecular function), whereas untreated (Li0) 3×-TgAD mice displayed associations with “regulation of protein localization”; “cell migration”; and “protein biding” (respectively). The network of genes related with neuron-glial interactions was further analyzed in 3×-TgAD treated with lithium, indicating statistically significant effects for “cellular organization” (*biological process*); “neuronal projection” (*cellular component*); and “cytoprotection and restoration of homeostasis” (*molecular function*). Results for the three GO domains were similar for Li1 and Li2. No statistically significant effects were found for Wt-Li1 (in all GO domains) and for Wt-Li2 on *molecular functions*.

**TABLE 3:** Over-represented (*p* < 0.05) gene ontology (GO) domains and related functions indicative of neuron-glia interactions.

Model:	Gene ontology domains and related functions
**(A) STRING dataset**	
Biological process	Trans membrane-ephrin receptor activity Glycolytic process Ephrin receptor signaling pathway
Cellular component	Postsynaptic specialization, intracellular component Phosphopyruvate hydratase complex
Molecular function	Transmembrane-ephrin receptor activity Ephrin receptor binding Neurotrophin receptor binding
**(B) Wt, Li0**	
Biological process	Golgi vesicle transport Endoplasmic reticulum to Golgi vesicle—mediated transport Cellular aminoacid metabolic process
Cellular component	Golgi-associated vesicle
Molecular function	Catalytic activity, acting on a tRNA Cullin family protein binding Ligase activity, form ins carbon -oxygen bonds
**(C) Wt, Lil**	
Biological process	–
Cellular component	–
Molecular function	–
**(D) Wt, Li2**	
Biological process	Regulation of presynapse organization Presynapse organization Regulation of filipodium assembly Neuromuscular process controlling balance
Cellular component	Golgi- associated vesicle Cul2-RING ubiquitin ligase complex Cullin-RING ubiquitin ligase complex
Molecular junction	–
**(E) 3×Tg-AD, Li0**	
Biological process	Positive regulation of establishment of protein localization to telomere Positive regulation of protein localization to cell periphery
Cellular component	Cell cortex region Chaperonic—containing T-complex
Molecular function	Unfolded protein binding NADP binding Transmembrane-ephrin receptor activity
**(F) 3×Tg-AD, Lil**	
Biological process	Protein folding chaperone

*STRING dataset (A) was further submitted to the scrutiny of different study groups, i.e., wild-type (Wt) mice (B–D) and transgenic (3×Tg-AD) mice (E–G).*

## Discussion

In the present study, we combine *in silico* and *in vivo* techniques to show that chronic treatment with lithium at subtherapeutic levels modifies ontological pathways related to neuron-glia interactions in 3×-TgAD mice. We used a hypothetical (*in silico*) model to obtain an enriched network of functional protein-protein interactions, in the light of which experimental data obtained by proteomic analysis of hippocampal tissues from 3×-TgAD and Wt mice exposed or not to lithium were cross-examined with the aid of bioinformatics tools; therefore we were able to build profiles of protein-protein interactions (PPIs) in experimental and control groups. In this model, chronic lithium treatment was shown to modify the pattern neuron-glia interactions in all relevant GO domains related to hippocampal homeostasis in AD-transgenic mice as compared to controls. Our data reinforce that lithium delivers an array of neuroprotective effects, multi-modal in nature, that may oppose certain AD-related pathogenic mechanisms, as reported in distinct experimental models (Kerr et al., 2018; Cuello et al., 2019; Wilson et al., 2020). Evidence of the neurotrophic and protective effects of lithium has been consistently replicated in all levels of experimentation (Machado-Vieira, 2018; Hampel et al., 2019), ranging from *in vitro* to clinical studies, with implications that may apply to many neurodegenerative disorders, including AD (Forlenza et al., 2011, 2016, 2019). Of note, Li et al. (2020) showed in a recent publication that chronic, low-dose treatment with lithium also attenuates cognitive dysfunction and pathological changes in AD transgenic mice (Liu et al., 2020).

Glial cells constitute an important fraction of the mammalian brain, where microglia, astrocytes and oligodendrocytes altogether account for 33–66% of the total brain tissue mass (Azevedo et al., 2009; Herculano-Houzel, 2014). Neurons critically need the support of glial cells in order to maintain homeostasis (Verkhratsky et al., 2019), and the interplay between neurons and glial cells is essential to brain function—the former playing executive, and the latter, housekeeping roles. Astrocytes are complex, highly differentiated glial cells that outnumber neurons by over fivefold. They perform many important functions, such as regulation of the extracellular environment, metabolite supply and maintenance of the integrity and function of the blood-brain barrier. In fact, astrocytes are key components of the microvascular beds, along with endothelial cells and the basal lamina (Del Zoppo and Hallenbeck, 2000).

Many neurodegenerative disorders are caused by proteinopathies, where the accumulation and aggregation of toxic proteins trigger pathological events that ultimately lead to loss of function and neuronal death (Soto and Pritzkow, 2018). Abnormal function of glial cells has been shown to play an important role in the pathophysiology of these disorders, particularly in AD (Herculano-Houzel, 2014). Neuroinflammation, a secondary but not less important phenomenon in the pathophysiology of AD, is a complex process organized by several groups of glial cells in the CNS and peripheral immune cells. The cross talk between many groups of glial cells present in neuroinflammation is also a dynamic process. For instance, activated microglia has been shown to mediate the synaptic deficits that arise from the presence of Aβ in the AD brain (Fonseca et al., 2004). Microglial cells are sensitive (and responsive) to the presence of Aβ peptides in the neural milieu, and activated microglia contributes to synaptic deficits even before the actual formation of amyloid plaques (Henstridge et al., 2019).

In this regard, certain modifications in the GO domains appeared as relevant effects of lithium in the presence of AD pathology. In our study of neuron-glia interactions, we found relevant and statistically significant effects on the GO domain *cellular component* in 3×-TgAD treated with subtherapeutic lithium, namely “cytosolic ribosome,” “axonal cytoplasm,” and “neuron projection cytoplasm.” These results suggest that lithium acts on several molecular targets that might lead to increased synapse formation. Likewise, lithium treatment also induced changes in genes related to “postsynaptic specialization.” This seems a coherent effect, given that perisynaptic glial cells regulate potassium homeostasis and modulate extracellular pH, in addition to expressing multiple membrane receptors that are sensitive to neurotransmitter signaling, therefore allowing a synergistic action with synaptic function, including those triggered by changes in intracellular calcium concentrations (Bateman et al., 2012; Rivera and Butt, 2019). Also, in the study by Wilot et al. (2004), lithium has also been shown to increase neuronal metabolism and neuronal projection in an experimental model of neurodegeneration (Wilot et al., 2004). In our study, when addressing the GO domain *molecular function*, we found evidence of changes in pathways related to “protein intermediates” and “forming polymers of unwanted aggregates”; abnormalities in these processes have been shown to be present in the pathogenesis of many neurodegenerative diseases, including AD (Pérez-Palma et al., 2014). We further found that chronic lithium treatment was associated with “increased misfolded proteins” generated in distinct cellular compartments (e.g., cytoplasm, nucleus, and endoplasmic reticulum), which are removed by the ubiquitin and autophagy proteasome system (Ciechanover and Kwon, 2015) and represent important repair mechanism in nerve cells. Our set of data also indicate effects on the GO domains *biological process* and *cellular component* which seem to be implicated with the “regulation of intermediate filaments” in the hippocampus. A recent study by Rivera and Butt (2019) showed that biological pathways related to astrocyte function are modified by lithium, leading to changes in extracellular matrix (ECM) regulatory enzyme lysyl oxidase (LOX) and peroxisome proliferator-activated gamma receptor (PPAR-γ) (Rivera and Butt, 2019).

Another finding within the GO domain *molecular function* was the effect on regulation of the ephrin (Eph) family of receptors. Ephrin’s complex initiates bidirectional signaling;, i.e., Eph receptors also act as ligands, and ephrin ligands also act as receptors (Pasquale, 2005). Eph signaling participates in different physiological processes, as well as cell migration and axonal growth, topographic mapping, axonal fasciculation and neurovascular formation in the developing nervous system (Mellitzer et al., 1999; Erskine et al., 2017), which is critically related to astrocyte function. Interestingly, this effect of lithium on Eph receptors was corroborated in a recent (unpublished) study conducted in our lab addressing transcripts from primary cultures of rat hippocampal neurons and cultures of human neuroprogenitor cells.

In conclusion, our results indicate that lithium modifies ontological pathways that probably underlie its neuroprotective responses. These effects are presumably expressed through neuronal projection pathways, regulation of glial cell activity, neuroinflammation and changes in misfolded proteins. We acknowledge, as a limitation of the present study, that we did not perform a thorough validation of the ontological data, i.e., addressing mRNA and/or protein expression related to the genes that constitute the ontological pathways that were shown to be affected by lithium. Nonetheless, the present set of data may support future studies addressing the role with neuron-glia interaction and neuroinflammation in the pathophysiology of AD and other neurodegenerative diseases, and the disease-modification potential of lithium treatment.

## Data Availability Statement

The original contributions presented in the study are publicly available. The datasets generated for this study are publicly available. This data can be found here: http://www.peptideatlas.org/PASS/PASS01638.

## Ethics Statement

The animal study was reviewed and approved by the local ethics committee (CAPPesq n°1293/09) in accordance with Directive 2010/63/EU.

## Author Contributions

NR and VD: participation in the collection, preparation of samples, ontological analysis, and preparation of the manuscript. RT and HB: preparation of samples, cleaning of data, and preparation of the manuscript. OF: ontological analysis and preparation of the manuscript. All authors contributed to the article and approved the submitted version.

## Conflict of Interest

The authors declare that the research was conducted in the absence of any commercial or financial relationships that could be construed as a potential conflict of interest.

## Footnotes

1
www.integratedproteomics.com

2
http://bioinfo.lbhc.hcancer.org.br/interactomegraph
