# Corrigendum: Neuronal–Glial Interaction in a Triple-Transgenic Mouse Model of Alzheimer's Disease: Gene Ontology and Lithium Pathways

**DOI:** 10.3389/fnins.2021.726983

**Published:** 2021-09-23

**Authors:** Nicole Kemberly R. Rocha, Rafael Themoteo, Helena Brentani, Orestes V. Forlenza, Vanessa De Jesus Rodrigues De Paula

**Affiliations:** ^1^Laboratório de Psicobiologia (LIM23), Departamento e Instituto de Psiquiatria, Hospital das Clinicas HCFMUSP, Faculdade de Medicina, Universidade de São Paulo, São Paulo, Brazil; ^2^Laboratorio de Neurociencias (LIM27), Departamento e Instituto de Psiquiatria, Hospital das Clinicas HCFMUSP, Faculdade de Medicina, Universidade de São Paulo, São Paulo, Brazil

**Keywords:** lithium, gene ontology, triple-transgenic model of Alzheimer's disease, hippocampus, neuronal-glial interaction

In the original article, we neglected to include the funder **FAPESP**, **2020/15145-8** to **Orestes V. Forlenza**.

The authors apologize for this error and state that this does not change the scientific conclusions of the article in any way. The original article has been updated.

## Publisher's Note

All claims expressed in this article are solely those of the authors and do not necessarily represent those of their affiliated organizations, or those of the publisher, the editors and the reviewers. Any product that may be evaluated in this article, or claim that may be made by its manufacturer, is not guaranteed or endorsed by the publisher.

