# Supplementary figures and images for: Neuronal–Glial Interaction in a Triple-Transgenic Mouse Model of Alzheimer’s Disease: Gene Ontology and Lithium Pathways
Source: Front Neurosci. 2020 Dec 1;14:579984. doi: 10.3389/fnins.2020.579984 (PMC7737403; doi:10.3389/fnins.2020.579984)

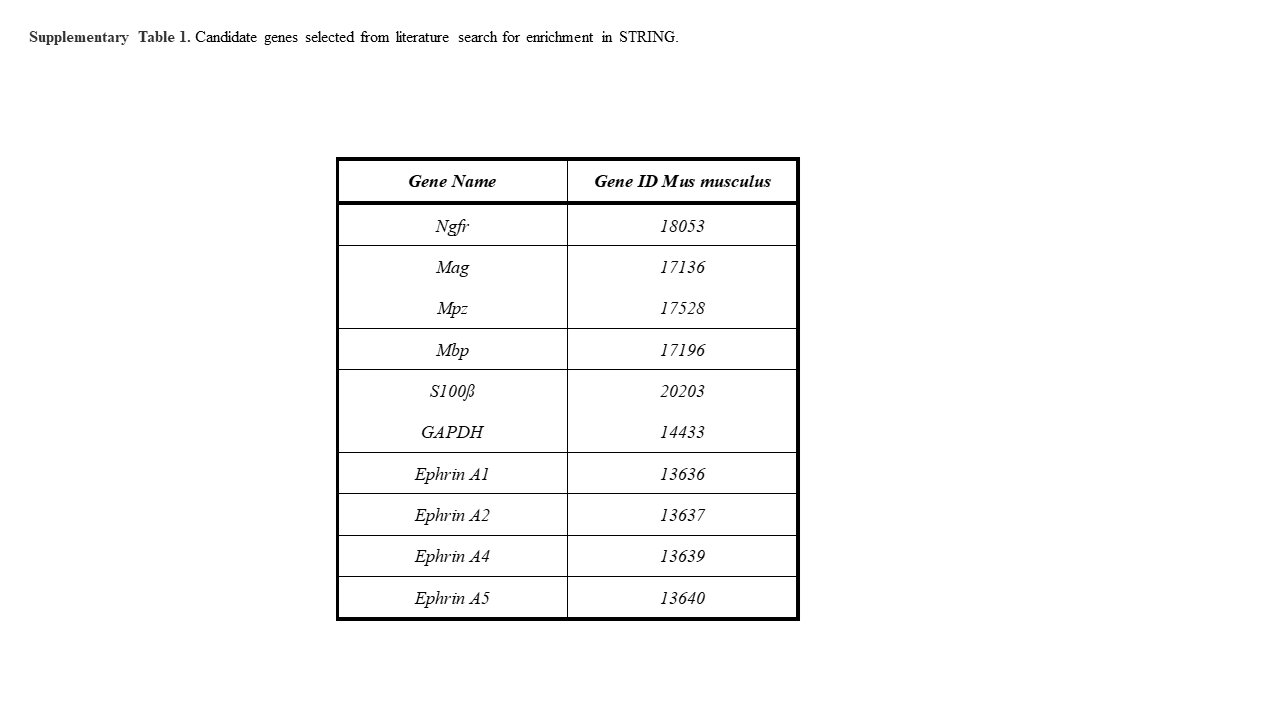

Supplement: Supplementary file 1 [file Image_2.TIF]

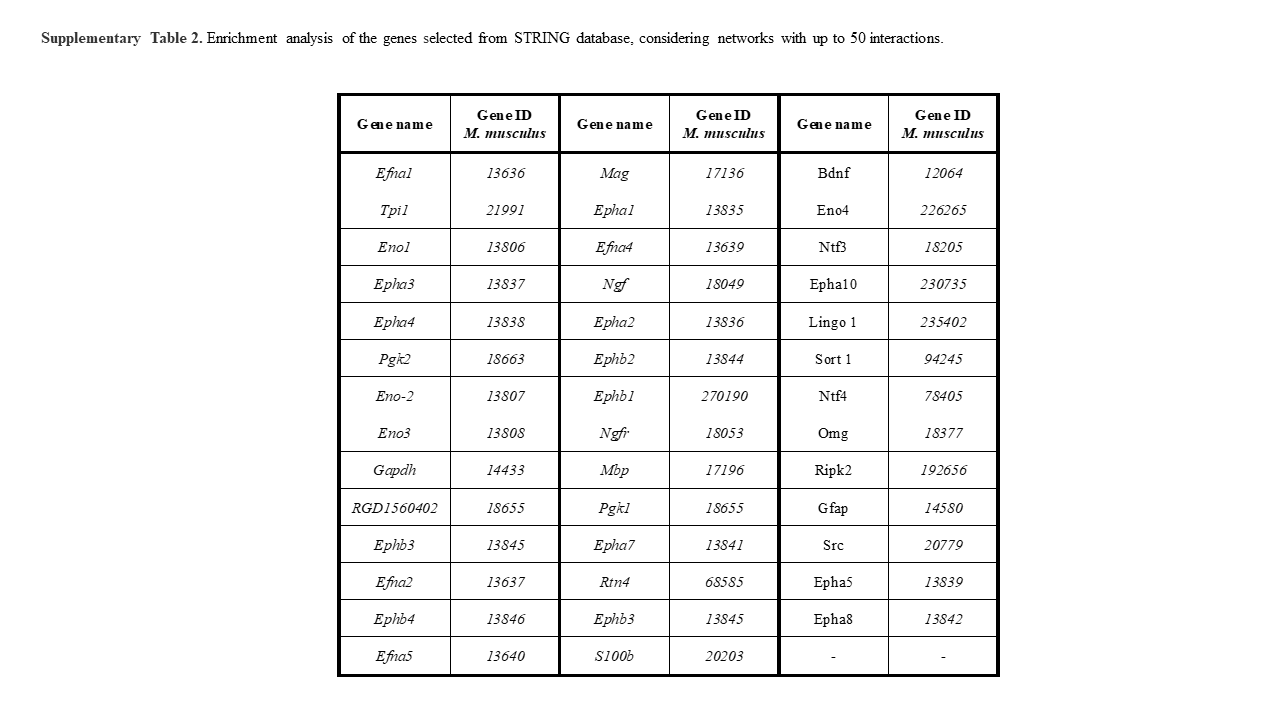

Supplement: Supplementary file 2 [file Image_1.TIF]
